# Memorializing Women on the Move: A register of migrant women landmarks in Europe

**DOI:** 10.12688/openreseurope.17052.1

**Published:** 2024-06-04

**Authors:** Maija Ojala-Fulwood, Bénédicte Miyamoto, Marie Ruiz, Heidi Martins, Fiona Eva Bakas, Veronika Čapská, Frigren Pirita, Lívia Prosinger, Igor Lyman, Victoria Konstantinova

**Affiliations:** 1 Tampere University, Tampere, Pirkanmaa, 33100, Finland; 2 Universite Sorbonne Nouvelle, Paris, Île-de-France, 75012, France; 3 Universite de Picardie Jules Verne, Amiens, Hauts-de-France, 80000, France; 4 Centre de Documentation sur les Migration Humaines, Dudelange, Luxembourg, 3481, Luxembourg; 5 Lusofona University, Lisbon, 1749-024, Portugal; 6 Czech Academy of Sciences, Prague, Czech Republic; 7 University of Turku, Turku, Southwest Finland, 28100, Finland; 8 Institute of Political History, Budapest, 1114, Hungary; 9 Berdyansk State Pedagogical University, Zaporizhzhia, 69000, Ukraine

**Keywords:** Migration Studies, Gender Studies, Women’s History, Minorization, Political Landscape, Public Memory, Toponomy, Mapping

## Abstract

This dataset was developed by COST Action 19112 Women on the Move (WEMov), which engages in unveiling women migrants' presence and participation in the construction of Europe. The dataset was built as a register of toponyms and monuments in the political and public landscape in Europe – such as street names, school names and parks, as well as statues and memorials – that celebrate women migrants. With the dataset we want to discover how women migrants are remembered and what kind of landmarks present these individuals who have had an episode of migration for a variety of reasons. Moreover, our aim is to make these landmarks and the stories of women migrants visible by presenting the results of the dataset in an interactive map on our website.

At the moment, the dataset includes 1000 landmarks. The collection of data was based on voluntary work of scholars and students from over 40 different European countries. We have aimed for broad geographical coverage; however, some areas are better represented than others due the nature of data collection. The collection of data is an ongoing process and therefore the dataset in Nakala repository, to which this data note refers, presents the situation in July 2023. Updated versions of the dataset will be made available in Nakala and we will download new landmarks to our interactive map on a regular basis.

The selected landmarks and migrant trajectories feature cross-community or cross-cultural migration. They show both typical and exceptional forms of mobility and present women of different age, profession, social status and migration status. This intersectionality of the project and the dataset highlights not only the richness of these landmarks and their value for scholarship but also the wide spectrum of migrant women and their contribution to society.

## Plain language summary

The dataset shows landmarks in memory of migrant women. These landmarks are the result of political decisions at one point in history to honour these women. The landmarks are tangible and denominated, available in the public space, such as buildings, streets, parks, monuments or gravestones. This collection of data shows how all these landmarks keep the memory of these migrant women alive in daily life. In the dataset we have 1000 landmarks from all over Europe. You can view them on our website.

## Introduction

The dataset Register of Migrant Women Landmarks has been developed as part of the COST Action Network 19112 Women on The Move. The idea of the dataset is to list European landmarks established in the memory of women who migrated in their lives. We registered not only street names but the vast array of urban toponyms, from monuments to varied urban infrastructure such as nurseries and hospitals, bridges and parks, as well as natural odonyms. According to our knowledge, this is the first time anyone has tried to map memorializations related to migrant women in the public sphere. With the register we explore what kind of migrant women landmarks exist and where they are located, mapping the presence of their stories in the cultural and political life of Europeans, as well as assessing the relative quantity of these specific landmarks associating notable women and stories of migration. The dataset makes these memorials and the stories of these migrant women visible and accessible and serves as a first sample analyzed in the ORE article ‘Materiality of Memorialisation: Mapping Women Migrant’s Landmarks in Europe’. It is the first data made available from an ongoing project collecting these landmarks, which are regularly transferred an interactive map that is available on the networks’ website (see Underlying data ( Bakas *et al.*, 2023))

## Methods

The main aim of the landmark data table is to provide the variables of each occurrence in Europe of migrant women’s landmarks. The 1000 rows of landmarks are sorted in columns of variables comprising landmark categories, geo-coordinates, address, country, short description, the identified woman’s occupation, and her standardized names and biographical dates.

For the selection of landmarks, the following criteria were applied:

- We recorded sixteen different categories of commemorative landmarks: burial (cemetery, mausoleum, tomb...); cultural venue (cinema, library, museum, theatre...) educational venue (school, university...); former residence; green space (garden, promenade, square...); housing (flat, council homes...); institute (foundation, research center...); monument (bust, statue...); medical venue (hospital, retirement home…); natural site (bay, lake, waterfall...); plaques (murals, stepping stones); religious venues (church, mosque, synagogue...); sports venue (stadium, swimming pool); street (avenue, boulevard, road...); temporary installation; transport infrastructure (airport, bridge, bus stop, station...).
- All landmarks had to represent a woman or women with a significant episode of migration in their history. In some cases, landmarks with family, married couple or a collective were included if the migrant women had played a significant role and were specifically identified by name or by representation.
  The individual migration trajectory should include cross-community or cross-cultural migration, if not cross-border migration. Short distance mobilities within the same cultural sphere, like moving house within a city or between same-nation cities were excluded.
- If there were several landmarks for one woman these would all be included. The landmarks could belong to the same category; for example, a number of streets dedicated to the same person, or represent different types of landmarks, such as street names, buildings and statues.
- All the landmarks recorded are located in Europe, and we did not place any historical boundaries to the selection of landmarks, or only record landmarks denoting European women.

For the selection of landmarks, the following methods were used:

To harvest the data, we mined a corpus of academic bibliographies, surveys, indexes and lists on toponyms and gender, as well as the collection of public information notices such as municipal and town hall minutes, local newspapers articles covering toponym news, and national registers and inventories of cadastral databases (with country specific geoportals such as the Romanian ANCPI geoportal, the Walloon Inventaire Centralisé des Adresses et des Rues (ICAR) or the French IGN Géoportail). We then effected a selection by identifying significant migration episodes in the women-based toponyms of the corpus. These results were then cross-referenced for the appearance of these women’s names in geolocalisation web services (such as OpenStreet Map, Google Maps or MapQuest) which included a wider range of toponyms. Landmarks identified were entered by a first contributor to a Google form and were then checked for conformity of data by a separate contributor before it was included on the landmarks register spreadsheet. This demanded extensive interdisciplinary cooperation within the COST network and allowed for outcome-oriented student workshops.

## Ethics and consent

Ethical approval and consent were not required.

## Data Availability

Nakala: The Microsoft Excel spreadsheet dataset showing the harvesting of landmarks as completed on July 2023 is available on Nakala DOI: https://www.doi.org/10.34847/nkl.28cf9j6q ( Bakas *et al.*, 2023) Interactive map on landmarks available on Map of Women Migrants’ Landmarks | WEMov (womenonthemove.eu) Data are available under the terms of the Creative Commons Attribution 4.0 International license (CC-BY 4.0)
