# Supplementary material for: Memorializing Women on the Move: A register of migrant women landmarks in Europe
Source: Open Res Eur. 2024 Jun 4;4:109. [Version 1] doi: 10.12688/openreseurope.17052.1 (PMC11380071; doi:10.12688/openreseurope.17052.1)
Supplement: Peer review history

# Reviewer report: Reviewer response for version 1

**DOI:** 10.21956/openreseurope.18426.r42670

**Published:** 2024-09-12

**Authors:** Susan Beth Rottmann

**Affiliations:** 1 Ozyegin University, Istanbul, Turkey

This is an excellent dataset of over 1000 landmarks. It is a wonderful teaching resource as well as a collection of interesting material for scholars working on migrant women, urban geography and history. The categories of commemorative landmarks are comprehensive and the interactive map is user-friendly. The methods and materials used are sound and can easily be cross-checked by others.

Are sufficient details of methods and materials provided to allow replication by others?

Yes

Is the rationale for creating the dataset(s) clearly described?

Yes

Are the datasets clearly presented in a useable and accessible format?

Yes

Are the protocols appropriate and is the work technically sound?

Yes

Reviewer Expertise:

Migration, gender, citizenship, food studies, anthropology, belonging

I confirm that I have read this submission and believe that I have an appropriate level of expertise to confirm that it is of an acceptable scientific standard.

# Reviewer report: Reviewer response for version 1

**DOI:** 10.21956/openreseurope.18426.r42668

**Published:** 2024-09-06

**Authors:** Kris Clarke

**Affiliations:** 1 University of Helsinki, Helsinki, Finland

This is a very innovative study that challenges the normalized ways that cityscapes are viewed and experienced. The aim of the article is to develop a database, not to provide a comprehensive literature review. The empirical data is extensive, visually interesting, and well-organized. This is important research and further discussion of the meaning of the data will be welcome in future research.

Are sufficient details of methods and materials provided to allow replication by others?

Yes

Is the rationale for creating the dataset(s) clearly described?

Yes

Are the datasets clearly presented in a useable and accessible format?

Yes

Are the protocols appropriate and is the work technically sound?

Yes

Reviewer Expertise:

Social work, decolonizing social work, harm reduction, HIV/AIDS, abolition

I confirm that I have read this submission and believe that I have an appropriate level of expertise to confirm that it is of an acceptable scientific standard.

# Reviewer report: Reviewer response for version 1

**DOI:** 10.21956/openreseurope.18426.r42278

**Published:** 2024-07-24

**Authors:** Umut Erel

**Affiliations:** 1 The Open University, Milton Keynes, England, UK

The article is clearly written, providing a clear rationale for the data set and explaining the methodology clearly. The data set itself is highly innovative and useable, contributing both to researchers' and students' of migration and gender's knowledge and research dissemination to non-academic audiences. 

 The only suggestion for improvement is that the current link does not lead directly to the map, but this link Map of women migrants’ landmarks – WEMov, Women on the move seems to lead to the data set. 

 An excellent resource and a clear article introducing it.

Are sufficient details of methods and materials provided to allow replication by others?

Yes

Is the rationale for creating the dataset(s) clearly described?

Yes

Are the datasets clearly presented in a useable and accessible format?

Yes

Are the protocols appropriate and is the work technically sound?

Yes

Reviewer Expertise:

gender, migration, racism, citizenship

I confirm that I have read this submission and believe that I have an appropriate level of expertise to confirm that it is of an acceptable scientific standard.

# Response

**Published:** 2024-07-26

**Authors:** Maija Ojala-Fulwood

Dear Umut Erel, Hope you are fine! Thank you so much for your review and drawing our attention that the link doesn't work properly. I will try to correct it as soon as possible. Have a nice summer! Greetings Maija
